# Underwater noise of traditional fishing boats in Cilacap waters, Indonesia

**DOI:** 10.1016/j.heliyon.2021.e08364

**Published:** 2021-11-11

**Authors:** Amron Amron, Rizqi Rizaldi Hidayat, Maria Dyah Nur Meinita, Mukti Trenggono

**Affiliations:** a Department of Marine Science, Jenderal Soedirman University, Purwokerto, 53123, Indonesia; b Department of Aquatic Resources, Jenderal Soedirman University, Purwokerto, 53123, Indonesia; c Centre for Maritime Bioscience, Jenderal Soedirman University, Purwokerto, 53123, Indonesia

**Keywords:** Noise, Traditional fishing boats, Frequency, Receive level, Cilacap waters

## Abstract

The characteristics of traditional fishing boats based on distance are very important to be studied as the main contributor to noise pollution in Cilacap waters. Therefore, this study aimed to determine noise intensity and frequency based on the distance for each traditional fishing boat (3, 5, and 10 Gross Tonage/GT). The results showed that these boats emitted noise with broadband frequency and peak receive levels of 137.6 dB re 1 μPa (3 GT fishing boat at 42.6 m). Furthermore, the noise characteristics were different for each type of ship due to differences in size, engine type, and operational speed. The receive level had the same decreased pattern based on the distance for each noise frequency but with a different intensity. Meanwhile, the noise frequency increased linearly based on the distance and was directly proportional to the pattern of change. Therefore, the higher the frequency, the faster the disappearance of the intensity with increasing distance.

## 1 Introduction

The practice of traditional fishing boats in Indonesia is used as the main transportation to optimize fisheries resources. It is made from simple technology and relies solely on the knowledge and experience of its makers. This is because the ship's tonnage is determined only based on the size (Praharsi et al., 2019). Furthermore, variation in tonnage causes differences in the ship's operating area following the national laws and regulations (Muawanah et al., 2018; Satria et al., 2018). In the context of current regulations, a traditional fishing boat is classified as a small-scale fishery, which ought to increase annually due to the optimization of resources (Halim et al., 2019). The increase in added value and market demand for catches from small-scale fisheries businesses has also triggered an increase in the number of traditional fishing boats (Adhuri et al., 2016).

However, the increased number of traditional fishing boats in Cilacap waters poses a dilemma to optimize and create pressure on fisheries resources. Shipping activity such as fishing is a major contributor to noise pollution in waters (McKenna et al., 2012; Soares et al., 2020). This has been evident in several studies, which prove that ships produce low-frequency noise with varying intensity (Dey et al., 2019; Lecchini et al., 2018; Marley et al., 2017; McCormick et al., 2019). The other sources are contributed by another anthropological activity (Blom et al., 2019; Fettermann et al., 2019), marine biota (Amron et al., 2018; Bejder et al., 2019; Caruso et al., 2017; Jézéquel et al., 2020), and physical processes (Buscaino et al., 2019; Paudel et al., 2020).

However, some vessels emit broadband frequency with relatively high intensity. This is closely related to the difference in the size of the ship, strength of the engine, and operational speed. Furthermore, small ships usually equipped with high-speed engines produce high frequencies (Brooker and Humphrey, 2016). On the contrary, larger ones have lower frequencies (McKenna et al., 2012). An increase in noise occurs when the ship is operated due to a combination of engine, propeller propulsion, and hydrodynamics (Zhang and Meng, 2018). The difference in the size of fishing vessels equipped with various types of engines causes the operating speed to be different. As a consequence, the noise produced by ships operating in Cilacap waters also varies. When this happens continuously, it will certainly impact the existence of aquatic biota since the ability to adapt also differs from one species to another (Popper and Hawkins, 2016; Soares et al., 2020).

Previous studies showed that anthropogenic noise from ships (broadband frequency with various intensities) affects the existence of marine life. The impact on marine mammals and fishes is in the form of stress (80–170 dB) (Rolland et al., 2012; Wright et al., 2011; Kight and Swaddle, 2011), leading to behavioral (up to 176 dB) (Halliday et al., 2017), physiological (up to 130 dB) (Codarin et al., 2009; Erbe et al., 2016) and other physiological effects (up to 170 dB) (Casper et al., 2013; Di Franco et al., 2020; Tougaard et al., 2015), population dynamics (high noise level) (Nabe-Nielsen et al., 2014), as well as damage to the hearing system (172–177 dB) (Slabbekoorn et al., 2010; Halliday et al., 2017). The sources and quantities of noise produced in Cilacap waters should be investigated due to the enormous impact on aquatic biota. However, there are limited studies on fishing vessels and noise intensity detection based on frequency. The impact of the noise of various ships on marine life was demonstrated more than two decades ago. Therefore, this study aims to determine the noise characteristics of frequency and intensity and changes based on distance in several types of traditional fishing boats: 3, 5, and 10 gross tonnages (GT).

## 2 Methods

### 2.1 Recording's site and system

**Figure 1:**
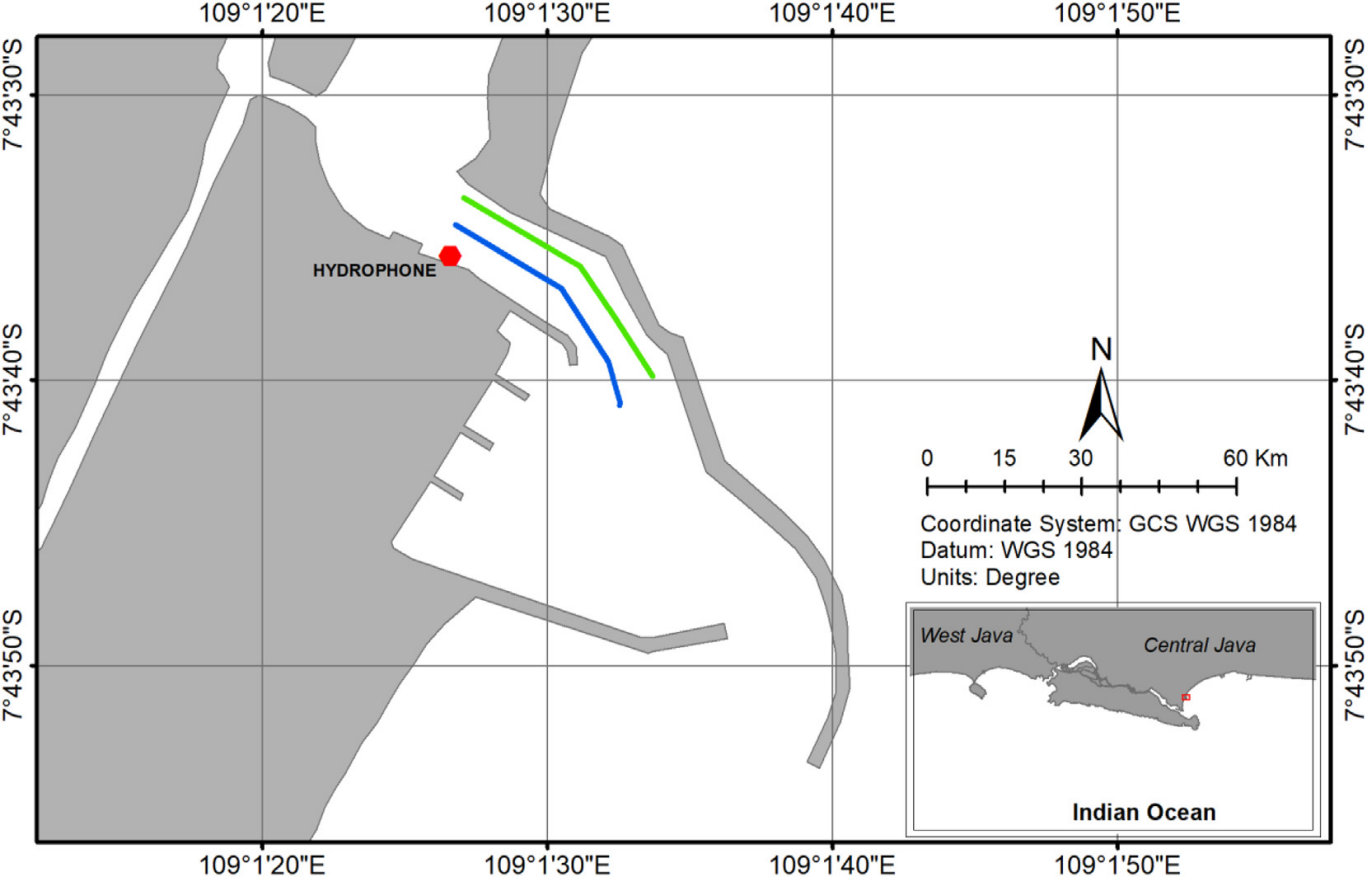
Recording's site. Solid lines represent to inner lane (blue) and the outer lane (green).

Noise emissions from 53 individuals for three types of traditional fishing boats (3, 5, and 10 GT; Figure 1) which pass the lane of Cilacap Fishing Port on September 6–8, 2019, were recorded using a calibrated omnidirectional hydrophone (Sea Phone SQ26-08; sensitivity -194 dB re 1 V.μPa^−1^, 20 Hz to 45 kHz flat response, and 25 dB gain). The hydrophone was installed in KP. Napoleon 33 (fishery patrol boat) that tethered in 7°43′35.13″ S and 109° 1′25.09″ E and deployed approximately 1.5 m under the sea surface (surface-based system). It was connected to a sound recorder (16-bit, 44,100 Hz sampling rate, and stored in WAV file). The recording's site was characterized by water depths ranging from 4.6 m low to 5.9 m high tide, and the seabed was made of clayey mud. Traditional fishing boats in the inner and outer lanes were recorded using an HD CCTV camera (1080 MP). The ship variations for each type were not significantly different in size, engine type, or operational speed. Furthermore, the departure and returning time of individual vessels to fishing activities was recorded twice a day. The closest recorded distance of the ship was 42.6 m from the recording's site. Meanwhile, the farthest distance was when CCTV recorded the ship, and its sound was recorded by hydrophone. Both sound and video recordings were synchronized, connected to Zoom H1n digital flash recorder, and displayed to an LCD monitor. Sound and video recordings were conducted continuously during the study.

### 2.2 Data analysis

Receive level (RL), and frequency of noise from each of the 5 individual vessels were determined using envelope and power spectral density (PSD) analysis. Sounds *S*(*t*) were recorded in volts and then converted to pressure, *P*(*t*) in μPa based on the time-domain (t). This was conducted using the following equation $P\left(t\right)=S\left(t\right)\times 10^{\frac{-G}{20}}\times D\times 10^{\frac{-SH}{20}}$, and *RL* (*t*) in dB re 1 μPa =20logP(t), where *G* (dB) is the recorder gain (here *G* = 25 dB), *D* is a constant for the dynamic response of the recorder (1.4 V for this model) and *SH* is the sensitivity of the hydrophone. Before the analysis, the recorded data was filtered using a high pass filter (500 Hz) and noise reduction caused by electronics or the environment. Meanwhile, the type of vessel that emits noise was analyzed based on video recordings. The ship's distance to the deployed hydrophone was a projection of the vessel's movement recorded by CCTV camera to the map and validated based on the speed. In addition, the pattern of changes in intensity based on time recording for each vessel was analyzed from the PSD of each frequency (1, 5, 10, and 15 kHz) by quadratic interpolation. The changes were conducted based on distance using simple linear regression, and a similar predictive model was used to determine the pattern of frequency changes for each group (<1, 2–3, and >4 kHz).

## 3 Results and discussion

### 3.1 Noise characteristics

The noise characteristics for RL and peak frequency vary based on differences in a traditional fishing boat (Table 1). The highest intensity was produced by 3 GT fishing boats, where the RL reached 137.6 dB re 1 μPa. With almost similar distance to the receiver position, RL of noise for 5 and 10 GT fishing boats reached 131.2 and 134.2 dB re 1 μPa, respectively. These variations were closely related to the difference in source level (SL), ship size, engine type, and strength, as well as the operational speed of the vessels. The 3 GT fishing boat emitted the highest noise intensity because it was equipped with a 20 HP gasoline-fueled outboard engine. With a small size and equipped engine power, it can travel at speeds up to 3.1 m s^−1^. The high operational speed causes the resulting noise intensity to be high due to the combination of engine, propeller, and hydrodynamics. It was different from the 5 GT fishing boat, which can only operate at a speed of 1.9 m s^−1^ leading to decreased noise intensity. In other cases, a 10 GT fishing boat equipped with a different engine (diesel-fueled inboard engine) and a power of 120 horsepower (HP) produced a higher noise intensity than a 5 GT type. Despite having the highest engine power, the ship's operational speed of only 0.9 m s^−1^ influenced the receive level of this vessel to be lower than the 3 GT fishing boat. Therefore, the intensity of noise produced by the ship was more dominantly influenced by the operational speed and other factors such as engine type and strength.

**Table 1:** Noise characteristics for a variety of traditional fishing boats.

Type of traditional fishing boat	Peak receive level (dB re 1 μPa)	Peak frequency (Hz)
3 GT (8.25 m of length and 1.2 m of width) with gasoline-fueled outboard engine (20 HP and 3.1 m s^−1^ of speed operation)	137.6	1,100
5 GT (11.00 m of length and 2.6 m of width) with the diesel-fueled inboard engine (45 HP and 1.9 m s^−1^ of speed operation)	131.2	1,886
10 GT (13.50 m of length and 2.8 m of width) with the diesel-fueled inboard engine (120 HP and 0.9 m s^−1^ of speed operation)	134.2	1,632

In contrast to RL, a 5 GT fishing boat produced the highest peak frequency that reached 1,886 Hz. However, it had the highest peak frequency that can be heard at 8 kHz. The 3 and 10 GT fishing boats have a frequency range that can reach the audible sound threshold (20 kHz). Their peak frequency was lower, which is only 1,110 and 1,632 Hz, respectively. Furthermore, the gasoline-fueled outboard engine type produced higher noise frequency than the diesel-fueled inboard engine. The power of the engine contributed greatly to this condition. Also, the wide range of noise frequencies produced by the 3 GT fishing boat was more reasonable because the ship's operating speed was higher. Because of this, the sound produced was more varied due to the combination of engine, propeller, and hydrodynamics. The variation in frequency produced by fishing vessels 10 GT was caused by the larger size and tonnage of the boat. Therefore, the frequencies produced by each source (engine, propeller, and hydrodynamics) were also more varied.

The various characteristics of noise from ships in the Cilacap waters are closely related to the high potential of the resources, which has been a particular incentive for local fishermen to optimize them as a source of income. The occurrence of illegal fishing, which has increased dramatically in Indonesian waters, has posed a threat to fisheries management (Satria et al., 2018). The condition also forces fishermen to increase the number of fishing fleets and compete in utilizing existing resources. Technological limitations required them to use makeshift in traditional boats for fishing (Praharsi et al., 2019). It was operated with a machine and was equipped with simple fishing gear (Halim et al., 2019). Therefore, there is a need to address an ecosystem approach to fisheries management (EAFM) to use sustainable resources (Muawanah et al., 2018).

The increase in the number of traditional fishing boats to optimize the use of fisheries resources has its implications. Different vessels with various sizes, engine types, and operating speeds contribute to the variation in the underwater noise produced. The 3 GT fishing boat was equipped with a gasoline-fueled outboard engine that dominated the effort in these waters. It can produce noise with a wider frequency range and a higher intensity compared to other types. This is because the type of engine used was a 2-stroke with high cavitation, which results in higher noise than other types with the same power (McCormick et al., 2019). Furthermore, small ships produce higher noise because they are equipped with high-speed engines and propellers (Brooker and Humphrey, 2016). Despite having lower spectral levels, 5 and 10 GT fishing boats also contribute to environmental noise in the waters. On the contrary, larger vessels produce low-frequency noise due to lower engine and propeller RPMs (McKenna et al., 2012). These differences are closely related to the speed, size, and load of the ship (Trevorrow et al., 2008), type of engine, propulsion system and propeller, and the sound mechanism (Hildebrand, 2009).

### 3.2 Noise intensity

**Figure 2:**
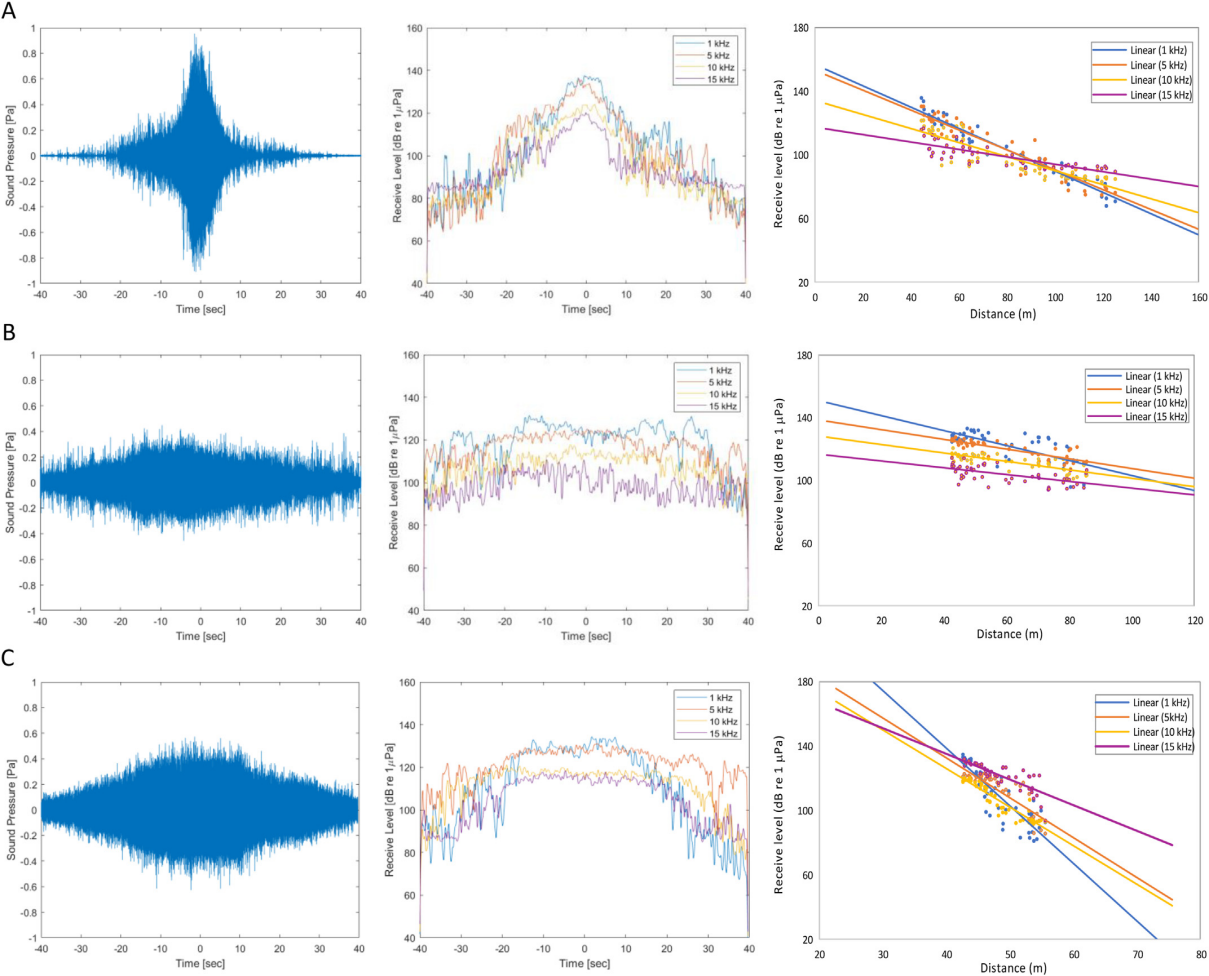
Noise intensity of traditional fishing boats; SPL (left) and RL for certain frequencies based on the time and distance (center and right). (A)–(C) represent 3, 5, and 10 GT, respectively.

Figure 2 shows further differences in noise intensity for each variety of traditional fishing boats. Generally, the changes in intensity vary based on the ship's type and movement (Figure 2, left). Following the waveform recorded by the receiver, the sound pressure level (SPL) increases since the noise was detected. In addition, it reaches the peak when in the closest distance with the receiver and then decreases as the ship moves away. Despite having the same pattern, the intensity and rate of change differ from one type of vessel to another. These include the highest SPL and the increased change pattern of noise intensity seen on 3 GT fishing boats (Figure 2A, left). This is inseparable from the ship's speed and is higher than others since the intensity of the SPL was also high. The rapid movement of the vessel as a consequence of such speed causes the distance with the receiver to change rapidly. The longer the ship's distance with the receiver, the lower SPL due to the presence of sound absorption, which may lead to increased transmission loss (TL). Furthermore, a similar reason was also directed to 5 and 10 GT fishing boats, where noise intensity also changes based on recording time due to changes in the distance (Figure 2B and 2C, left). The 5 GT fishing boat had a slower change in intensity even though the speed is higher than that of 10 GT. This is because the SPL is lower as a result of decreased engine power that complements the ship.

Figure 2 (center and right) shows the changes in RL of fishing boat noise for each frequency (1, 5, 10, and 15 kHz) based on the time and distance. Generally, it had the same pattern at the recording time changes as a consequence of distance. However, the intensity of noise seen is different when the ship approaches the closest distance to the receiver. RL for noise frequency of 1 kHz was not much different from 5 kHz. Therefore, the noise frequency of 10 kHz was similar to 15 kHz in both 3, 5, and 10 GT fishing boats. The noise frequencies up to 5 kHz can be detected well by receiving levels above 126 dB re 1 μPa. Meanwhile, the RL above 10 kHz was below 126 dB re 1 μPa. As the ship moves away from the receiver, there was no significant intensity difference for each frequency. This is because the existing RL was a combined intensity of broadband frequency.

The noise changes of each frequency as a function of the time and distance for 3 GT fishing vessels are shown in Figure 2A (center and right). At the closest distance (42.6 m), the RL was the highest intensity (137.6, 130.4, 121.5 and 115.9 dB re 1 μPa for frequency at 1, 5, 10 and 15 kHz respectively). As the vessel moves away 52.7 m (10 s), the noise intensity decreases proportionally for each frequency. The decrease continues to occur when it moves away to 75.2 m (20 s), the RL was the intensity of the broadband frequency with ranges to 105.0 dB re 1 μPa. Different intensity changes were shown by 5 GT fishing boat, where RL did not have significant changes to a distance of 57.5 m (20 s) with an intensity of 125.1 and 112.6 dB re 1 μPa for frequency up to 5 kHz and above 10 kHz, respectively (Figure 2B, center and right). Meanwhile, the decrease in intensity in all frequency groups was significant due to the increasing distance of the ships, and the resulting intensity was a combination of several frequencies (broadband). The same pattern changes occurred on the 10 GT fishing boat, where the intensity change was seen as the vessel moved away 61.8 m (20 s), as shown in Figure 2C (center and right).

The difference in the changes of noise intensity for each of these frequencies showed that variations in ship size, engine's type and power, and operational speed influenced the difference in SL. Noise intensity decreased due to changes in the propagation distance (Halliday et al., 2017; McKenna et al., 2012). The decrease in intensity for each frequency is a TL quantity dependent on absorption and beam spreading. Furthermore, the absorption coefficient is related to the frequency and aquatic media, such as temperature, salinity, and depth (Krishnaswamy and Manvi, 2015). The higher the frequency, the greater the change since the absorption coefficient increases. Meanwhile, the TL caused by beam spreading is a consequence of the sound propagation from the source (omnidirectional), which is spherical shape, so that TL=20logR (Etter, 2018).

Fluctuations in TL described by RL for each frequency based on distance are caused by the interference of sound waves called Lloyd's Mirror effect (LME). In shallow waters, omnidirectional noise from ships can cause interference from the sound path and those reflected by the surface and bottom of the water. LME depends on the depth, slant range, and frequency of the sound source and the receiver (Pereira et al., 2020). A deeper sound source forms a larger slant range. Conversely, a deeper receiver forms a smaller slant range. The slant range formed helps to determine the distance between the two sound paths. Therefore, it is possible to increase or decrease the received sound amplitude at a certain distance (Etter, 2018). The effect of frequency on LME is seen below 1 kHz, and the impact is lost at high frequencies above 10 kHz with a spherically distributed pressure (Audoly and Meyer, 2017).

### 3.3 Noise spectra

**Figure 3:**
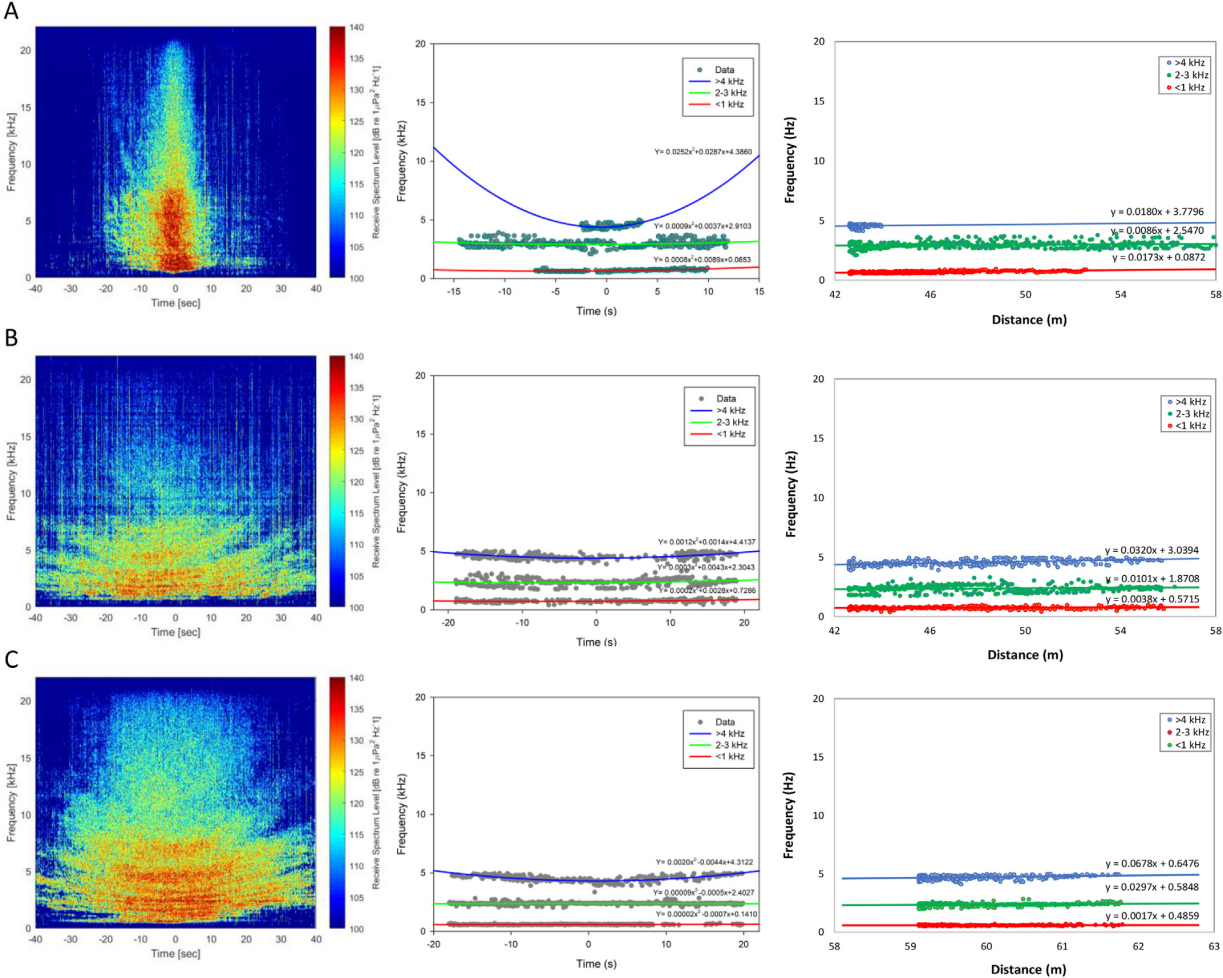
Noise spectrogram of traditional fishing boats based on the time (left and center) and the distance (right). (A)–(C) represent 3, 5, and 10 GT, respectively.

Figure 3 shows the fishing boat noise spectra based on the ship's travel time, representing the distance. Spectra noise appears to vary based on the type of fishing boat, where the frequency of 3 GT had a wider range and experiences faster loss of spectra based on distance compared to the 5 and 10 GT (Figure 3, left). However, the frequency had increased quadratically based on the distance, and the pattern of settlement varies. This variation was due to differences in the source frequency and operating speed, ship types, and engines. The smaller 3 GT fishing boat, equipped with a 20 HP gasoline-fueled outboard engine, can be operated at a higher speed, influencing the spectra noise to be produced in wider broadband frequencies and disappear faster with recording time (Figure 3A, left). Also, 5 and 10 GT fishing boats, both equipped with a diesel-fueled outboard engine, have the same spectra pattern. Small differences may be observed in the frequency range, and the increasing pattern based on the distance, where the 5 GT fishing boat has a smaller range and the changing pattern is slightly larger than the 10 GT (Figure 3B dan 3C, left). This occurs due to differences in engine power and operational speed of the two types of vessels.

Figure 3 (center and right) shown a further change in frequency for each vessel based on time recording and distance. Generally, the group (<1, 2–3, and >4 kHz) experienced a quadratic increase along with the time and linear increase based on the distance. The higher the frequency, the clearer is the reduction pattern for the three types of fishing vessels. In 3 GT, the frequency changes were seen to be lower in the <1 kHz group, with a quadratic coefficient of 0.0008 (based on the time) and a linear coefficient of 0.0073 (based on the distance) (Figure 3A, center and right). On the contrary, the pattern of increasing frequency slightly changed in the 2–3 kHz group, where the linear coefficient increased to 0.0086. The changes were observed in the higher group, where the coefficient was above 0.0180 when the noise frequency was >4 kHz. Even though it had the same tendency, the coefficient of increasing frequency was lower on the 5 GT fishing boat (Figure 3B, center and right). In the same frequency group, the linear coefficients for this type of vessel only ranged from 0.0017, 0.0297, and 0.0678, respectively. This value was still higher than the linear coefficients for each group produced by 10 GT fishing boats, which were 0.0038, 0.0101, and 0.0320 belonged to the frequency group of <1, 2–3, and >4 kHz, respectively (Figure 3C, center and right).

Therefore, the variation of the source frequency as a representation of ship and engine type, as well as operation speed influenced differences in receive frequency and change patterns. In contrast to intensity, the frequency had increased due to changes in the ship's position and the receiver when it is operated (Fillinger et al., 2011; Sutin et al., 2010), as a consequence of the doppler effect (Ahmad et al., 2018). The existence of noise with certain spectra can even be detected at certain distances generated by the traditional fishing boat. This has the potential to threaten the existence of marine life, both fish and mammals. Some of the threats from ship noise to biota were in the form of behavioral disturbances, physical damage, and even causing death (Popper and Hawkins, 2016).

Previous studies on aquatic biota (see Dey et al., 2019; Lecchini et al., 2018; Marley et al., 2017; McCormick et al., 2019) reminds the existence of a traditional fishing boat in Cilacap waters should be managed in such a way that the available resources remain sustainable. Several countries and regions have formulated regulations regarding the limits of noise which allowed for shipping-making operating vessels to comply with these regulations. Several international agreements that are concerned with underwater noise impacts were the European Union (EU) Marine Strategy Framework Directive (MSFD), International Whaling Commission (IWC), Convention on the Conservation of Migratory Species of Wild Animals (CMS), Arctic Council's Protection of Arctic Marine Environment (PAME), International Maritime Organization (IMO), Helsinki Commission (HELCOM), OSPAR Commission (OSPAR), International Union for the Conservation of Nature (IUCN), Convention on Biological Diversity (CBD), and UN-Oceans Resolution (Lewandowski and Staaterman, 2020). Unfortunately, the relevant regulations are not regulated in Indonesian waters, so fishing vessels can operate freely without binding monitored. When this continues, it was predicted that marine life in the waters would be increasingly threatened along with the increasing use of ships as a main for marine transportation.

## 4 Conclusion

It is reasonable to conclude that the variation of traditional fishing boats equipped with various engine types and strengths influenced the characteristics of the noise emitted. Furthermore, noise intensity based on frequency can be detected at a certain distance with a decreasing pattern. The higher the frequency, the faster the disappearance of the intensity along with increasing distance. Also, the constrained number and type of vessels observed resulted in the limitation of information on the characteristics of the ship's noise. However, this initial information is very important and requires a comprehensive study related to the ship's noise and their impact on marine biota as an initial effort to mitigate noise pollution in Indonesian waters.

## Declarations

### Author contribution statement

Amron Amron: Conceived and designed the experiments; Performed the experiments; Analyzed and interpreted the data; Contributed reagents, materials, analysis tools or data; Wrote the paper.

Rizqi Rizaldi Hidayat: Conceived and designed the experiments; Performed the experiments; Analyzed and interpreted the data; Wrote the paper.

Maria Dyah Nur Meinita: Conceived and designed the experiments; Analyzed and interpreted the data; Wrote the paper.

Mukti Trenggono: Conceived and designed the experiments; Performed the experiments; Wrote the paper.

### Funding statement

This work was supported by Indonesian Ministry of Research, Technology and Higher Education (Kemenristekdikti).

### Data availability statement

Data included in article/supplementary material/referenced in article.

### Declaration of interests statement

The authors declare no conflict of interest.

### Additional information

No additional information is available for this paper.
